# Reliability and validity of the Finnish version of the AO spine PROST (patient reported outcome spine trauma)

**DOI:** 10.1186/s13018-025-06021-6

**Published:** 2025-06-23

**Authors:** Leevi A. Toivonen, Jussi P. Repo, Hannu Kautiainen, Said Sadiqi, Marko H. Neva

**Affiliations:** 1 Department of Orthopedics and Traumatology, Tampere University Hospital and Tampere University, Elämänaukio 2, PB 272, Tampere, 33101 Finland; 2 Primary Health Care Unit, Kuopio University Hospital, Kuopio, Finland; 3 Folkhälsan Research Center, Helsinki, Finland; 4 Combined Neurosurgical and Orthopedic Spine Program, Vancouver General Hospital, University of British Columbia, Vancouver, Canada; 5 Department of Orthopaedics, University Medical Center Utrecht, Utrecht, the Netherlands

**Keywords:** AO spine PROST, Spine trauma, Spine fracture, Patient reported outcome, EQ-5D-3L, Oswestry disability index

## Abstract

**Purpose:**

To translate AO Spine PROST (Patient Reported Outcome Spine Trauma) into Finnish and explore its psychometric properties in a Finnish spine trauma population.

**Methods:**

Patients were enrolled at an academic level-I trauma center. Score distribution, floor and ceiling effects, and missing items were explored for content validity. Internal consistency, concurrent validity, and reproducibility were explored with Cronbach’s α, exploratory factor analysis, Spearman correlation tests, and Intraclass Correlation Coefficients (ICCs).

**Results:**

Translation was performed using established guidelines. A sample of 110 patients was enrolled. Score distribution skewed toward higher values, but no floor and ceiling effects were present. Response rates were excellent for all items except for Work/study (60%). Factor analysis indicated two possible dimensions in PROST, explaining 75.3% of the total variance. For PROST total score, correlations were strong with EQ-5D-3 L (*r* = 0.77 [95% confidence interval: 0.69–0.84]) and ODI (*r*=-0.89 [0.92–0.84]). Test-retest reliability was excellent with ICC = 0.86 (0.76–0.91). Calculation of the PROST score without the Work/study item appeared valid (ICC 0.99 [0.98 to 1.00]).

**Conclusions:**

The Finnish version of AO Spine PROST is a reliable and valid measure for spine trauma outcomes. We recommend it for clinical practice and research to reduce the current controversies in spine trauma care.

**Supplementary Information:**

The online version contains supplementary material available at 10.1186/s13018-025-06021-6.

## Introduction

Measuring function and quality of life has become central in monitoring quality of medical care [1, 2]. Traditionally, outcomes of treatment were measured binary as survival or death. Over the years, with improvements in healthcare and the majority of trauma patients surviving their injuries, the focus has shifted to more comprehensive outcome measures. More recently, patient-reported outcome measures (PROMs) have been developed to capture patients’ perspectives on their disease and treatment. The available disease-specific measures for spine patients focused on functional limitations related to degenerative pathologies and appeared inadequate for patients sustaining spine trauma [2]. These measure patient’s current limitations in performance, whereas in trauma outcomes, the change from pre-injury levels is relevant. The most used spine measure, the Oswestry Disability Index (ODI), grades functional restrictions stemming from back pain [3]. However, spine trauma may deliver severe functional deficits without pain.

Under AO Spine Knowledge Forum Trauma, the AO Spine PROST (Patient Reported Outcome Spine Trauma) instrument was developed to address the above limitations [2, 4]. In contrast to traditional PROM instruments, it compares post-injury performance to pre-injury levels. It was first developed in Dutch [5] and has been thereupon translated and validated into several languages, including English [6], German [7], Slovak [8], and Nepalese [9]. The questionnaire consists of 19 items on different aspects of everyday life, each being answered on a numeric rating scale ranging from 0 to 100. In each item, the respondent compares their current function to their pre-injury levels.

AO Spine PROST has been or is being translated in a total of 17 languages. To the best of our knowledge, no translation is available in the Finnish language. The purpose of this study was to translate the AO Spine PROST into Finnish and evaluate its validity and reliability in a representative sample of a Finnish spine trauma population.

## Methods

### Translation process

The questionnaire was translated into Finnish by established principles [10]. First, two native Finnish speakers independently made a forward translation from English into Finnish. Second, the two translators merged their versions into one consensus version, considering potential needs for country-specific adaptations. Third, a bilingual professional translator naïve to the topic did a backward translation from Finnish to English. Fourth, an expert committee from the study group reviewed the translation process and its different versions and thereafter introduced the Finnish version. Fifth, the consensus version was tested with ten participants who were asked to fill in feedback on paper if they found any difficulties in understanding the questions or if they had any suggestions for modifications to the questionnaire.

### Subjects

Participants were enrolled from the population of a level-I trauma center via two routes. First, we screened for eligible patients who were enrolled into a prospective follow-up of thoracolumbar spine fractures that began in April 2023. Second, hospital records were queried for patients who presented for spine trauma between 2022 and 2024 and had missed the offer to be enrolled into the prospective follow-up. Recruitment started with more recent injuries and continued until the targeted sample size of over 100 patients was reached. The target was based on the COSMIN risk of bias checklist for conducting psychometric studies [11].

Adults (≥ 18 years) with fracture at the cervical, thoracic, lumbar, or sacral (spinopelvic type) spine, and no or at most moderate spinal cord injury (American Spinal Injury Association [ASIA] Impairment Scale C to E) were included. Exclusion criteria included para- or tetraplegia (ASIA Impairment Scale A or B), severe polytrauma (Injury Severity Score, ISS > 15), and cognitive status insufficient for completing the surveys.

### Data collection

All participants gave their written, informed consent. Subjects of the prospective follow-up completed questionnaires through REDCap (Research Electronic Data Capture tool) hosted at the authors’ institution [12]. Their first follow-up responses at six weeks postinjury including The Oswestry Disability Index, EQ-5D-3 L, and AO Spine PROST were used in the validation study. Participants from the cross-sectional sample were mailed an information sheet, consent and questionnaire forms that they returned with a prepaid envelope.

### PROM instruments

*AO Spine PROST* (Online resource 1) consisting of 19 questions each grading a specific part of function on a numeric rating scale from 0 (no function) to 100 (pre-injury level of function). AO Spine PROST total score is calculated as the sum of all responses divided by the number of completed items.

*Oswestry Disability Index (ODI)* [3] consisting of 10 items resulting in a score from 0 (no disability) to 100 (maximal disability).

*EQ-5D-3 L* [13] being a generic health measure consisting of five questions and a visual analogue scale which can be converted into a single score ranging from − 0.594 (worst possible health) to 1 (perfect health).

For test-retest reliability evaluation, the first 47 enrolled patients were mailed the AO Spine PROST form a second time two weeks after their initial response. Thereafter, patients were mailed only one round of questionnaires to reduce respondents’ burden. Clinical data on patient demographics and injury details were collected from hospital records by authors. Ethics committee approval (R22113) was obtained prior to inception of the prospective follow-up. An institutional study permit (R24561) was granted for the cross-sectional part of the study in accordance with Finnish legislation.

### Statistical methods

Descriptive data on the subjects were presented using means with standard deviation (SD), medians with interquartile range (IQR), or counts with percentages. Floor and ceiling effects were explored, defined as being present when the proportion of patients scoring minimum or maximum points outnumbered 15%, respectively [14, 15]. Internal consistency of the AO Spine PROST total score was estimated by calculating Cronbach’s α with bias corrected bootstrap 95% confidence intervals (95% CI). Exploratory factor analysis with iterated principal-factor method for factoring and promax-rotated factor loadings on correlation matrix was performed to assess dimensionality in AO Spine PROST. The strategies used to extract the number of factors were the Kaiser criterion which determines that components with eigenvalues lower than one should be excluded, and the screen test of Cattell criteria. Reproducibility or agreement for measurements (test-retest reliability) was assessed with Intraclass Correlation Coefficients (ICCs) from a one-way random model, coefficient of repeatability (CR), or by Bland and Altman plot (with limits of agreement (LOA) for paired observations). CR expresses the expected maximum difference expected with 95% probability between paired observations. Concurrent validity was explored with Spearman correlation coefficients between AO Spine PROST and ODI and EQ-5D-3 L with Sidak-adjusted (multiplicity) probabilities. The possible non-linear relationships between AO Spine PROST and ODI or EQ-5D-3 L were modeled using restricted cubic splines with three knots at the 10th, 50th, and 90th percentiles. Models were adjusted for age and sex. The statistical significance of the correlation was estimated with Sidak-adjusted probabilities. We also compared EQ-5D-3 L values of participants to values in the age and sex-matched general Finnish population [16]. Study design and reporting adhered to the COSMIN Study Checklist for Patient-Reported Outcome Instruments and the STROBE checklist [11]. All analyses were performed using STATA software, version 18.0 (StataCorp LP, College Station, TX).

## Results

### Translation and cross-cultural adaptation

The translation process encountered no difficulties. Its phases are detailed in Online resource 1. On the pretesting phase, no difficulties were reported, nor any modifications were proposed. One respondent described their health state in general. Thus, no adaptations were made for the final Finnish version of AO Spine PROST (Online resource 1).

### Patient characteristics

A consecutive cohort of 26 eligible patients were enrolled from the prospective follow-up. To the cross-sectional part, 197 eligible patients were invited, and 84 (47%) of them consented. Therefore, the total study sample consisted of 110 patients. Detailed demographic and injury characteristics are described in Table 1.

**Table 1:** Baseline characteristics of the study population. If multiple spine fractures were present, the index injury is described

Variable	Measurement *N* = 110
Age, mean (SD) [Range]	57 (17) [20–81]
Male sex, n (%)	70 (64)
Patients with comorbidities, n (%)	58 (53)
Working status, n (%)	
Working	51 (46)
Retired	54 (49)
Unemployed	2 (2)
Not known	3 (3)
Injury mechanism, n (%)	
MVA	25 (23)
Fall from height	31 (28)
Sports/recreational	17 (15)
Low energy	30 (27)
Other	7 (6)
Anatomic location, n (%)	
Cervical	26 (24)
Thoracic	40 (36)
Lumbar	43 (39)
Sacral (spinopelvic type)	1 (1)
Fracture classification (AO Spine), n (%)	
A	74 (67)
B	32 (29)
C	4 (4)
ASIA Impairment scale, n (%)	
C	3 (3)
D	10 (9)
E*	97 (88)
Surgical management, n (%)	
No	73 (66)
Yes, anterior approach	7 (6)
Yes, posterior approach	27 (25)
Yes, combined approach	3 (3)

MVA, motor vehicle accident; SD, standard deviation; ASIA, American Spinal Injury Association; Patients without spinal cord injury also included in E group

### Content validity

**Fig. 1:**
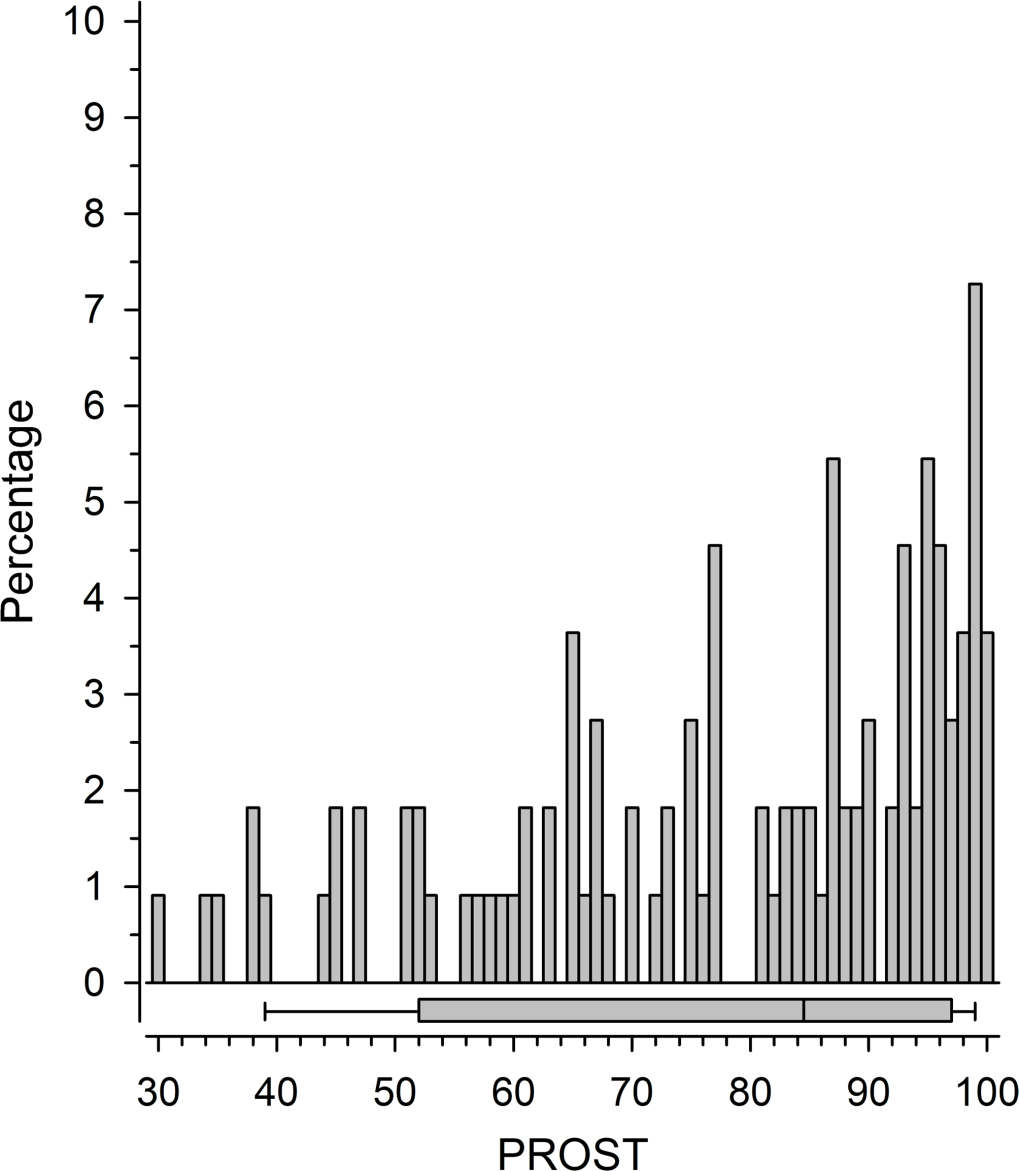
Histogram of the AO Spine PROST total scores in the study population demonstrates their obvious rightward skewed distribution. Box plot shows median with interquartile range, and whiskers indicate 5th and 95th percentiles

The median (IQR) time from injury to PROST completion was 9 (2, 22) months. The total PROST score distribution was skewed toward higher scores (Fig. 1), but the ceiling effect in the total score was not reached (maximum points 4%).

Excellent response rates (97–100%) were observed in all items except in “Work/study” (60%) which the survey text instructs to skip if the respondent were not working/studying before the injury (Table 2).

**Table 2:** Response rate, floor and ceiling effects in PROST items

AO Spine PROST item	Response rate (%)	Mean (SD)	Median (IQR)	Floor, *n* (%)	Ceiling, *n* (%)
1. Household activities	100	75 (26)	82 (62, 99)	1 (1)	22 (20)
2. Work/study	60	70 (30	75 (50, 99)	2 (3)	14 (21)
3. Recreation and leisure	99	65 (29)	70 (50, 90)	2 (2)	10 (9)
4. Social life	100	82 (23)	90 (70, 100)	0 (0)	32 (29)
5. Walking	100	80 (24)	90 (70, 99)	0 (0)	27 (25)
6. Travel	100	78 (26)	90 (60, 99)	0 (0)	22 (20)
7. Changing posture	100	78 (21)	80 (69, 97)	0 (0)	21 (19)
8. Maintaining posture	100	76 (22)	80 (60, 97)	0 (0)	16 (15)
9. Lifting and carrying	99	68 (30)	76 (50, 95)	1 (2)	16 (15)
10. Personal care	100	86 (19)	95 (80, 100)	0 (0)	30 (27)
11. Urinating	100	92 (16)	99 (90, 100)	1 (1)	46 (42)
12. Bowel movement	99	93 (16)	99 (95, 100)	0 (0)	54 (50)
13. Sexual function	97	78 (30)	90 (62, 100)	4 (4)	28 (26)
14. Emotional function	99	84 (22)	95 (72, 100)	0 (0)	31 (28)
15. Energy level	100	78 (23)	90 (63, 99)	0 (0)	24 (22)
16. Sleep	100	83 (19)	90 (70, 99)	0 (0)	26 (24)
17. Stiffness of your neck and/or back	100	70 (24)	80 (50, 90)	0 (0)	9 (8)
18. Loss of strength in your arms and/or legs	100	79 (23)	89 (65, 99)	0 (0)	22 (20)
19. Back and/or neck pain	100	73 (23)	80 (51, 90)	0 (0)	15 (14)

### Internal consistency

Exploratory factor analysis was performed to explore possible dimensionality in AO Spine PROST (Table 3). It was conducted without item 2 with lower response rate (60%). Most items loaded high on factor 1 while “Personal care”, “Urinating”, and “Bowel movement” loaded high on factor 2. Internal consistency was excellent between each factor and the PROST total score (Cronbach α = 0.97 for factor 1, and Cronbach α = 0.88 for factor 2). The two factors explained 75.3% of the total variance in AO Spine PROST.

**Table 3:** Exploratory factor analysis for the possible dimensionality in AO spine PROST

AO Spine PROST item	Factor 1	Factor 2
1. Household activities	0.86	
3. Recreation and leisure	0.87	
4. Social life	0.72	
5. Walking	0.70	
6. Travel	0.75	
7. Changing posture	0.86	
8. Maintaining posture	0.83	
9. Lifting and carrying	0.90	
13. Sexual function	0.62	
14. Emotional function	0.62	
15. Energy level	0.71	
16. Sleep	0.65	
17. Stiffness of your neck and/or back	0.86	
18. Loss of strength in your arms and/or legs	0.76	
19. Back and/or neck pain	0.84	
10. Personal care		0.72
11. Urinating		0.92
12. Bowel movement		0.92
% of variance	66.2	9.1
Cronbach’s α (95% CI)	0.97 (0.96 to 0.98)	0.88 (0.77 to 0.94)

Coefficients with values < 0.5 not shown

Consistency between AO Spine PROST total score calculated with and without item 2 was excellent (ICC 0.99 [95% CI: 0.98 to 1.00]).

### Test-retest reliability

**Fig. 2:**
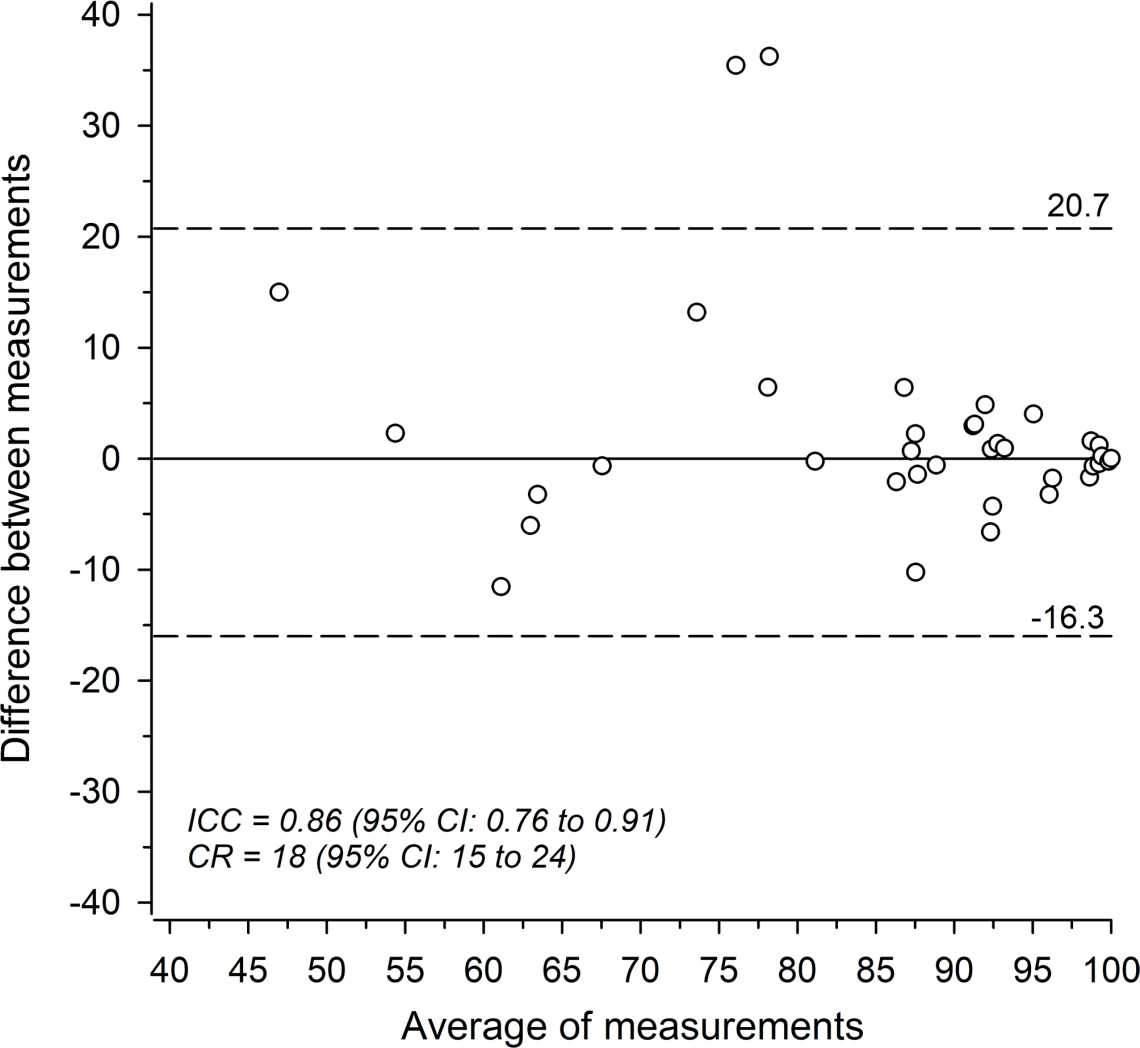
Bland-Altman plot showing the average of the PROST total score and the difference between the measurements. The dashed lines indicate the 95% limits of agreement. ICC indicates intraclass correlation coefficient; CR, coefficient of repeatability

AO Spine PROST was re-mailed to 47 participants two weeks after the completion of the first round. Of those, 39 (83%) completed the second round. Thereafter, subjects were only invited to participate in one round. Median (IQR) interval between measurements was 23 (16, 29) days. Figure 2 illustrates the agreement between measurements with Bland-Altman plot. Excellent reliability was observed in AO Spine PROST total score between measurements (ICC 0.86 [95% CI: 0.76 to 0.91]). Reliability was excellent in all items except in Changing posture, Stiffness of your neck or back, and Back or neck pain, where it remained good. ICC for single items ranged between 0.95 (0.91 to 0.97) for Sleep and 0.61 (0.36 to 0.75) for Back or neck pain.

### Construct validity

**Fig. 3:**
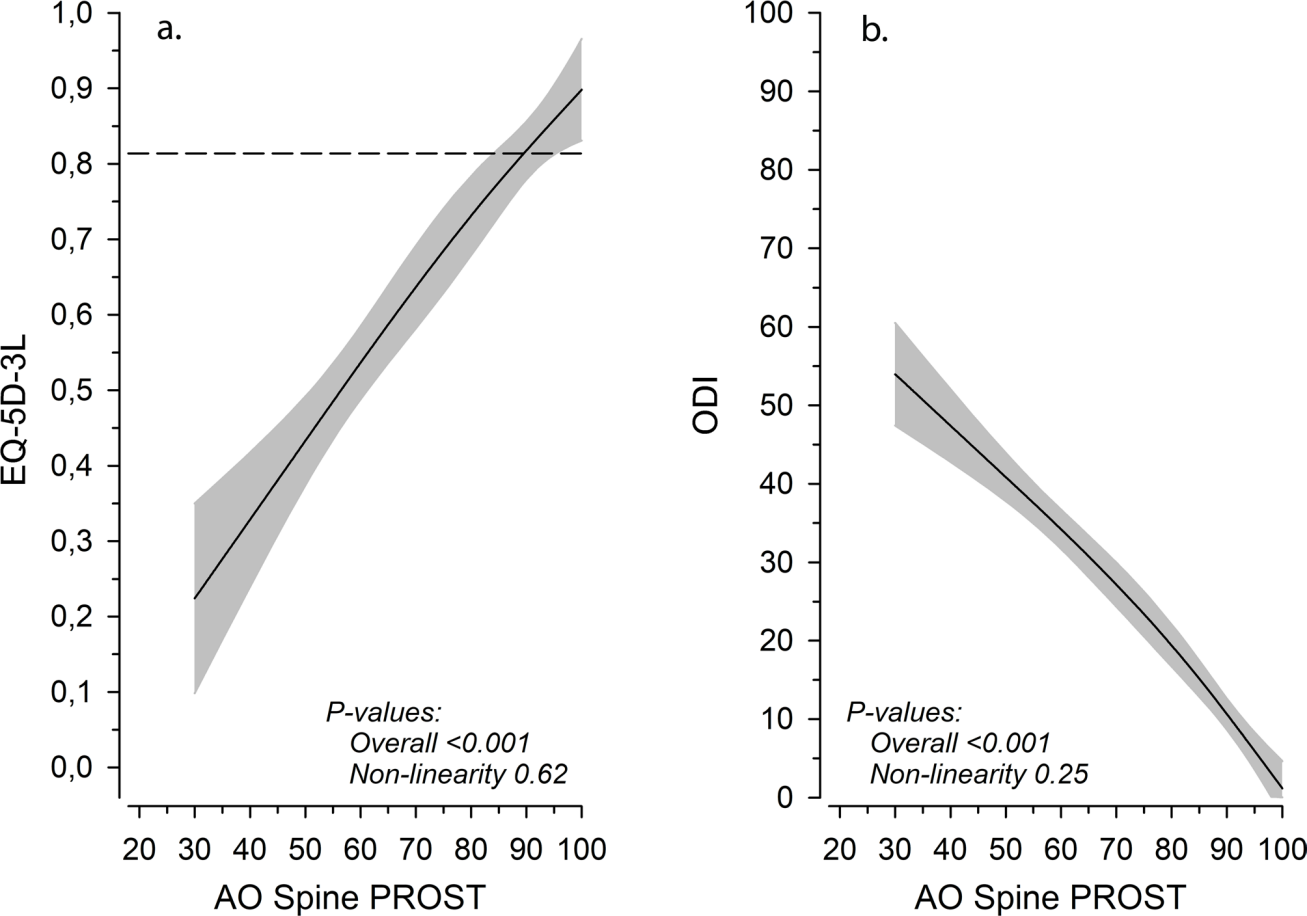
**A**, correlation between AO Spine PROST and EQ-5D-3 L, adjusted for age and sex, with 95% confidence intervals. The dashed line illustrates Finnish age- and sex-adjusted general population quality-of-life levels. **B**, correlation between AO Spine PROST and ODI, adjusted for age and sex, with 95% confidence intervals

There was a strong positive correlation (*r* = 0.77 [95% CI: 0.68 to 0.84]) between the AO Spine PROST total score and EQ-5D-3 L, and a strong negative correlation (*r* = -0.89 [-0.92 to -0.84]) between the AO Spine PROST total score and ODI. Both correlations were linear when adjusted for age and sex, as illustrated in Fig. 3. Correlations between distinct PROST items and EQ-5D-3 L and ODI scores are described in Table 4. The three most correlating items of AO Spine PROST with EQ-5D-3 L were Recreation and leisure, Lifting and carrying, and Walking. With ODI they were Lifting and carrying, Household activities, and Recreation and leisure.

**Table 4:** Correlations between AO spine PROST and EQ-5D and ODI

AO Spine PROST item	Equation 5D *r* (95% CI)	ODI *r* (95% CI)
1. Household activities	0.69 (0.57 to 0.78)***	-0.82 (-0.87 to -0.75)***
2. Work/study	0.70 (0.55 to 0.81) ***	-0.78 (-0.86 to -0.66) ***
3. Recreation and leisure	0.75 (0.65 to 0.82) ***	-0.82 (-0.87 to -0.75) ***
4. Social life	0.59 (0.45 to 0.70) ***	-0.66 (-0.75 to -0.54) ***
5. Walking	0.72 (0.61 to 0.80) ***	-0.79 (-0.85 to -0.71) ***
6. Travel	0.66 (0.54 to 0.76) ***	-0.73 (-0.81 to -0.63) ***
7. Changing posture	0.64 (0.51 to 0.74) ***	-0.72 (-0.80 to -0.61) ***
8. Maintaining posture	0.63 (0.50 to 0.73) ***	-0.76 (-0.83 to -0.67) ***
9. Lifting and carrying	0.74 (0.64 to 0.81) ***	-0.84 (-0.89 to -0.78) ***
10. Personal care	0.65 (0.52 to 0.74) ***	-0.73 (-0.81 to -0.63) ***
11. Urinating	0.32 (0.14 to 0.48) *	-0.38 (-0.53 to -0.20) **
12. Bowel movement	0.25 (0.06 to 0.42)	-0.28 (-0.44 to -0.10)
13. Sexual function	0.52 (0.36 to 0.64) ***	-0.76 (-0.83 to -0.67) ***
14. Emotional function	0.48 (0.31 to 0.61) ***	-0.55 (-0.67 to -0.40) ***
15. Energy level	0.63 (0.50 to 0.73) ***	-0.70 (-0.78 to -0.59) ***
16. Sleep	0.49 (0.33 to 0.62) ***	-0.64 (-0.74 to -0.51) ***
17. Stiffness of your neck and/or back	0.69 (0.58 to 0.78) ***	-0.80 (-0.86 to -0.72) ***
18. Loss of strength in your arms and/or legs	0.63 (0.51 to 0.74) ***	-0.76 (-0.83 to -0.66) ***
19. Back and/or neck pain	0.68 (0.56 to 0.77) ***	-0.75 (-0.82 to -0.65) ***
Total PROST score	0.77 (0.68 to 0.84) ***	-0.89 (-0.92 to -0.84) ***
Factor 1	0.77 (0.67 to 0.84) ***	-0.89 (-0.92 to -0.83) ***
Factor 2	0.59 (0.45 to 0.70) ***	-0.69 (-0.77 to -0.57) ***

Sidak-adjusted probabilities: *p < 0.05, **p < 0.01, ***p < 0.001

## Discussion

This study produced a Finnish version of AO Spine Patient Reported Outcome Spine Trauma questionnaire. It showed excellent psychometric properties in exploring spine trauma outcomes in a Finnish population.

The study sample can be regarded as a representative sample of spine fracture patients, half of the sample consisting of older individuals with low energy fractures. Distribution of fracture severity by AO Spine classification aligned with validity studies from the Netherlands [5] and North America [6]. The German validity study [7] comprised modestly more severe fractures and substantially more often surgical management. Although ceiling effects in single items are not relevant to score validity, we reported ceiling percentages in single items to describe AO Spine PROST distribution. The observed high ceiling percentages in the items Urinating (42%) and Bowel movement (50%) highlight the heterogeneity in spine trauma populations where patients sustaining milder injuries in general do not encounter urinary or bowel problems, rather limitations to functional activities in daily life. Also, many limitations are temporal as a great share of injuries has a favorable healing potential. In the present study, the interval from injury to the AO Spine PROST administration (median 9 months) may have shifted the results further rightward, as patients likely had already progressed from their greatest initial postinjury disabilities. Longer intervals may also trigger recall bias as patients have to compare their current state to distant baseline and they may have adapted to their new health state. Interestingly however, a recent study showed good reliability and validity results for AO Spine PROST in the long-term (median 94.5 months) follow-up [17].

Previous validation studies for AO Spine PROST have used quality-of-life measures for benchmarking concurrent validity. The current study is the first study where AO Spine PROST was compared both with a generic quality-of-life measure (EQ-5D-3 L) as well as a back-specific disability measure (ODI). Correlation was slightly greater with ODI, despite the fact that ODI works mainly by grading functional limitations stemming from back pain. This implies pain plays a role in posttraumatic limitations, as well, although spine trauma patients may suffer even greater impairment from functional deficits. Those functional impairments are the main focus of the PROST items, with the unique feature to measure the current performance as compared to preinjury functional levels. Our results demonstrated AO Spine PROST as a comprehensive measure for spine trauma outcomes. It appeared capable of capturing the essential aspects of spine trauma outcomes also across cultural boundaries–at least in the West, given no cross-cultural adaptations were needed for the Finnish population.

In line with the validation study on the Dutch and North American populations [5, 6], our findings indicated two factors in AO Spine PROST: the first factor describing outgoing activities and coping, and the second factor describing intimate activities. The majority of spine trauma patients do not sustain spinal cord injuries which may have detrimental effects on intimate functions such as urinary, bowel, or sexual impairments. Therefore, spine trauma outcomes in the majority could be analyzed without the second factor items. AO Spine PROST was first developed in patients with at most incomplete spinal cord injury [4]. Later, its applicability in complete spinal cord injury populations has been demonstrated, as well [18]. This however may change the role of the second factor in their outcomes, owing to impaired intimate functions with spinal cord injury.

An ideal score would consist of items with low nonresponse. In spine trauma populations where half of subjects are beyond working age, the item “Work/study” understandably yields nonresponses–especially when the survey instructs to skip the item when not applicable. We observed that in the Finnish spine trauma population, omitting the item “Work/study” from the AO Spine PROST total score did not result in reduced performance. This may be used in further developmental phases of the instrument.

### Limitations and strengths

The present study has several limitations. Subjects were enrolled from a single academic center, although we believe in Finland academic trauma centers capture a fairly population-based sample of spine trauma patients.

The relatively long interval between injury and measurement (median 9 months) could have resulted in higher scores reflecting the more advanced recovery stage but also triggered recall bias. Also, our test-retest interval (median 23 days) surpassed the common preference of two weeks [14], but longer interval from injury apparently reduced the risk of a change in the respondent’s condition between measurements. Patients with complete spinal cord injury or severe polytrauma were not included thus limiting the analysis of psychometric properties of AO Spine PROST to milder injuries. The rigorous translation and cross-cultural adaptation process guaranteed that the Finnish version has equivalent content validity to the original version of the AO Spine PROST. The sample size of the present study (*n* = 110) can be considered sufficient for conducting psychometric testing [11]. The sample in test-retest analysis (*n* = 39) is limited but we believe a larger sample would not have changed our results.

## Conclusion

The Finnish AO Spine PROST is a reliable and valid measure for spine trauma outcomes. We recommend it to be used in clinical practice and research to reduce the ongoing controversies in certain types of spine trauma care.

## Electronic supplementary material

Below is the link to the electronic supplementary material.

Supplementary Material 1

## Data Availability

Under Finnish law, sharing of datasets with person data is prohibited.
