# Supplementary material for: Reliability and validity of the Finnish version of the AO spine PROST (patient reported outcome spine trauma)
Source: J Orthop Surg Res. 2025 Jun 23;20:607. doi: 10.1186/s13018-025-06021-6 (PMC12183804; doi:10.1186/s13018-025-06021-6)
Supplement: Supplementary file 1 — Supplementary Material 1 [file 13018_2025_6021_MOESM1_ESM.pdf]

Supplementary Information for the article Reliability and Validity of the Finnish version of the AO Spine PROST (Patient Reported Outcome Spine Trauma)

Online resource 1 Translation process for the Finnish AO Spine PROST.

PROST items	Forward translation 1	Forward translation 2	Consensus translation	Back translation	Original
1	Kotiaskareet (kuten siivoaminen sisällä ja pihalla, pyykinpesu tai ruuan valmistaminen)	Kotityöt (kuten siivous sisällä tai ulkona, pyykinpesu tai ruuanlaitto	Kotiaskareet (kuten siivoaminen sisällä ja ulkona, pyykinpesu tai ruuanlaitto)	Household chores (e.g. cleaning inside and outside, doing the laundry or cooking)	Household activities (such as cleaning in and around the house, doing laundry or preparing a meal)
2	Työ/opinnot (jos et tehnyt töitä tai opiskellut ENNEN onnettomuutta, siirry seuraavaan kysymykseen)	Työ/opiskelu (jos et ollut töissä tai opiskellut ENNEN onnettomuutta, siirry seuraavaan kohtaan)	Työ/opiskelu (jos et ollut töissä tai opiskellut ENNEN onnettomuutta, siirry seuraavaan kysymykseen)	Working/studying (if you weren't working or studying BEFORE the accident, go to the next question)	Work/study (if you were not working or studying BEFORE the accident, please skip this question)
3	Virkistystoiminta ja vapaa-aika (kuten harrastukset tai liikunta)	Virkistys ja vapaa-aika (kuten harrastukset tai urheilu)	Virkistäytyminen ja vapaa-aika (kuten harrastukset tai liikunta)	Recreation and leisure (e.g. hobbies or sports)	Recreation and leisure (such as hobbies or sports)
4	Sosiaalinen elämä (kuten suhteiden ylläpitäminen perheen, ystävien ja tuttavien kanssa)	Sosiaalinen elämä (kuten yhteydenpito perheisiin, ystäviin ja tuttaviin)	Sosiaalinen elämä (kuten yhteydenpito perheeseen, ystäviin ja tuttaviin)	Social life (e.g. contact with family, friends and acquaintances)	Social life (such as maintaining relationships with family, friends and acquaintances)
5	Käveleminen (ilman apuvälineitä tai apuvälineiden kanssa)	Kävely (apuvälineen kanssa tai ilman)	Kävely (apuvälineen kanssa tai ilman)	Walking (with or without an assistive device)	Walking (with or without an aid)
6	Matkustus (kuten ajaminen itse, julkisten liikennevälineiden käyttö tai muu liikenneväline)	Matkustus (kuten autolla-ajo, joukkoliikenteen tai muiden liikkumistapojen käyttö)	Matkustus (kuten autolla ajaminen, joukkoliikenteen tai muiden liikkumistapojen käyttö)	Travelling (e.g. driving, using public transport or other modes of transport)	Travel (such as driving yourself, using public transportation or other means of transport)
7	Asennon vaihtaminen (kuten makaaminen, istuminen tai seisominen)	Asennonmuutos (kuten makuulle, istumaan tai seisomaan)	Asennon vaihtaminen (makuulle, istumaan tai seisomaan)	Changing positions (lying down, sitting down or standing up)	Changing posture (such as lying down, sitting or standing)
8	Asennon pitäminen (kuten makaaminen, istuminen tai seisominen niin kauan kuin tarpeen)	Asennon ylläpito (kuten makaaminen, istuminen tai seisominen niin pitkään kuin tarpeen)	Asennon ylläpitäminen (kuten makaaminen, istuminen tai seisominen niin pitkään kuin tarpeen)	Maintaining positions (e.g. lying, sitting or standing for as long as necessary)	Maintaining posture (such as lying down, sitting or standing, for as long as necessary)
9	Nostaminen ja kantaminen (kuten kauppakassin nostaminen tai lapsen kantaminen)	Nostaminen ja kantaminen (kuten ostoskassin nostaminen tai lapsen kantaminen)	Nostaminen ja kantaminen (kuten ostoskassin nostaminen tai lapsen kantaminen)	Lifting and carrying (e.g. lifting a shopping bag or carrying a child)	Lifting and carrying (such as lifting a bag of groceries or carrying a child)
10	Henkilökohtainen hygienia (kuten kylvyn tai suihkun ottaminen, wc:n käyttäminen tai pukeutuminen ja riisuutuminen)	Henkilökohtainen hygienia (kuten kylvyssä tai suihkussa käynti, vessassa käynti, pukeminen tai riisuminen)	Henkilökohtainen hygienia (kuten kylvyssä tai suihkussa käynti, vessassa käynti, pukeminen tai riisuminen)	Personal hygiene (e.g. taking a bath or a shower, going to the toilet, getting dressed or undressed)	Personal care (such as taking a bath or shower, using the toilet or dressing and undressing)

11	Virtsaaminen (pystytkö virtsaamaan; pystytkö pidättämään virtsaasi)	Virtsaaminen (pystytkö virtsaamaan; pystytkö pidättelemään)	Virtsaaminen (pystytkö virtsaamaan; pystytkö pidättämään virtsaa)	Urination (can you urinate; can you hold urine)	Urinating (are you able to urinate; can you hold your urine)
12	Suolen toiminta (pystytkö ulostamaan; pystytkö pidättämään ulostetta)	Suolen toiminta (pystytkö ulostamaan; pystytkö pidättelemän)	Suolen toiminta (pystytkö ulostamaan; pystytkö pidättämään ulostetta)	Bowel function (can you defecate; can you hold stool)	Bowel movement (are you able to have a bowel movement; can you hold your bowel movement)
13	Seksuaalinen toimintakyky	Sukupuolielämä	Seksuaalinen toimintakyky	Sexual functioning	Sexual function
14	Tunnekyky (kuten synkkyys, huoli tai ahdistavat tunteet)	Tunne-elämä (kuten synkät, huolestuneet tai ahdistavat tunteet)	Tunnekyky (kuten synkkyys, huoli tai ahdistavat tunteet)	Emotional functioning (e.g. gloominess, worry or feelings of anxiety)	Emotional function (such as gloomy, worried or anxious feelings)
15	Energiataso (kuten väsyneisyys tai voimattomuus)	Jaksaminen (kuten uupumus tai välinpitämättömyys)	Jaksaminen (kuten uupumus tai voimattomuus)	Stamina (e.g. exhaustion or lack of energy)	Energy level (such as fatigue or listlessness)
16	Uni (kuten tuntien määrä tai unenlaatu)	Uni (kesto ja laatu)	Uni (kesto ja laatu)	Sleep (duration and quality)	Sleep (such as number of hours and quality)
17	Jäykkyys niskassa ja/tai selässä (koskien häiriötä kokonaistoimintakyvyssä)	Niskan ja/tai selän jäykkyys (liittyen yleiseen toimintakyvyn puutteeseen)	Niskan ja/tai selän jäykkyys (liittyen yleiseen toimintakyvyn puutteeseen)	Stiffness of neck and/or back (relating to a lack of general functional ability)	Stiffness of your neck and/or back (in terms of disability in overall performance)
18	Voiman puuttuminen käsissä ja/tai jaloissa (koskien häiriötä kokonaistoimintakyvyssä)	Voimattomuus ylä- tai alaraajoissa (liittyen yleiseen toimintakyvyn puutteeseen	Voimattomuus ylä- ja/tai alaraajoissa (liittyen yleiseen toimintakyvyn puutteeseen	Lack of strength in upper and/or lower limbs (relating to a lack of general functional ability)	Loss of strength in your arms and/or legs (in terms of disability in overall performance)
19	Selkä- ja/tai niskakipu (koskien häiriötä kokonaistoimintakyvyssä)	Selkä- tai niskakipu (liittyen yleiseen toimintakyvyn puutteeseen)	Selkä- ja/tai niskakipu (liittyen yleiseen toimintakyvyn puutteeseen)	Back and/or neck pain (relating to a lack of general functional ability)	Back and/or neck pain (in terms of disability in overall performance)
Answer options	En ole TOIMINTAKYKYINEN	Olen TOIMINTAKYVYTÖN	Olen TOIMINTAKYVYTÖN	I am UNABLE TO FUNCTION	I am NON-FUNCTIONAL
	Toimin yhtä hyvin kuin ENNEN onnettomuutta	Toimintakykyni on sama kuin ENNEN onnettomuutta	Toimintakykyni on sama kuin ENNEN onnettomuutta	My functional ability is the same as BEFORE the accident	I function as well as BEFORE my accident
